# Preclinical evaluation of alternatives to oral immunotherapy for food allergies

**DOI:** 10.3389/falgy.2023.1275373

**Published:** 2023-10-03

**Authors:** Brandi T. Johnson-Weaver

**Affiliations:** Department of Pathology, Duke University Medical Center, Durham, NC, United States

**Keywords:** food allergy, immunotherapy, mouse models, immunotherapy route, immune modulation

## Abstract

The increasing food allergy incidence has led to significant interest in developing therapies for allergic diseases. Oral allergen-specific immunotherapy (OIT) is a recently FDA-approved therapeutic to treat peanut allergies. OIT utilizes daily allergen dosing to reduce allergic reactions to peanuts. However, there is diminished enthusiasm for daily OIT, potentially due to the strict regimen required to induce desensitization and the risks of severe adverse events. Thus, there remains a need for safe and effective food allergy treatments that are well-received by allergic individuals. Preclinical research studies investigate methods to induce allergen desensitization in animals and support clinical studies that address the limitations of current food allergy OIT. Because allergic reactions are triggered by allergen doses above an individual's activation threshold, immunotherapy regimens that induce allergen desensitization with lower allergen doses or without the requirement of daily administrations may expand the use of food allergy immunotherapy. Administering allergen immunotherapy by alternative routes is a strategy to induce desensitization using lower allergen doses than OIT. Several animal models have evaluated oral, sublingual, epicutaneous, and intranasal immunotherapy routes to treat food allergies. Each immunotherapy route may require different allergen doses, formulations, and treatment schedules to induce desensitization. This article will discuss scientific findings from food allergy immunotherapy animal studies that utilize various immunotherapy routes to induce allergen desensitization to support future clinical studies that enhance the safety and efficacy of allergen immunotherapy to treat food allergies.

## Introduction

Allergen-specific immunotherapy is emerging as a treatment for food allergies, which are estimated to affect 10% of the American population (1). Preclinical food allergy studies aim to induce desensitization and sustained unresponsiveness (SU) to allergens by identifying immunotherapy conditions that dampen immune responses that mediate allergic disease. This mini-review will discuss findings from animal immunotherapy studies that may be useful in developing effective immunotherapy regimens to treat human food allergies. Preclinical immunotherapy studies have generated preliminary support for clinical trials to evaluate the safety and efficacy of food allergy immunotherapy. This led to peanut oral immunotherapy (OIT) being approved for human use in the United States and Europe. Human OIT requires daily peanut dosing of up to 300 mg (2) and effectively desensitizes 79% of patients (3). Clinical allergen-desensitization modifies allergen-specific immune responses after therapy and increases the allergen dose required to induce an allergic reaction (4). Despite inducing desensitization, OIT can also cause adverse events (AE) during treatment, and SU is only achieved in 13%–50% of individuals after discontinuing therapy (5, 6). Thus, next-generation immunotherapy is required to reduce AEs and enhance SU.

One strategy to enhance OIT safety and efficacy is administering the immunotherapy formulation by an alternative route. Clinical studies are evaluating sublingual (SLIT) and epicutaneous (EPIT) immunotherapy as alternative therapies for food allergies (7, 8). SLIT and EPIT utilize 4 mg (7) and 250 μg (8, 9) of peanut, respectively, to induce desensitization, which is lower than the 300 mg peanut dose consumed during OIT (2). Lower allergen doses may contribute to the increased safety profile observed in SLIT and EPIT studies compared to OIT (10–12). Despite reducing therapy-mediated AEs, SLIT and EPIT are less effective than OIT for inducing desensitization. 70% of subjects who complete SLIT can tolerate at least 800 mg of peanut (7), and 37% of EPIT-treated subjects obtained a cumulative reactive dose of 3,444 mg (8, 9). In comparison, some OIT studies report 93%–100% of subjects tolerating greater than 3 g of peanut (4, 13). Varying immunotherapy safety and efficacy observed when different routes are used to administer peanut immunotherapy suggests the immunotherapy route is essential when developing immunotherapy regimens. However, the contribution of the immunotherapy route to modulating protective immunity against food allergies is not entirely understood.

**Figure 1:**
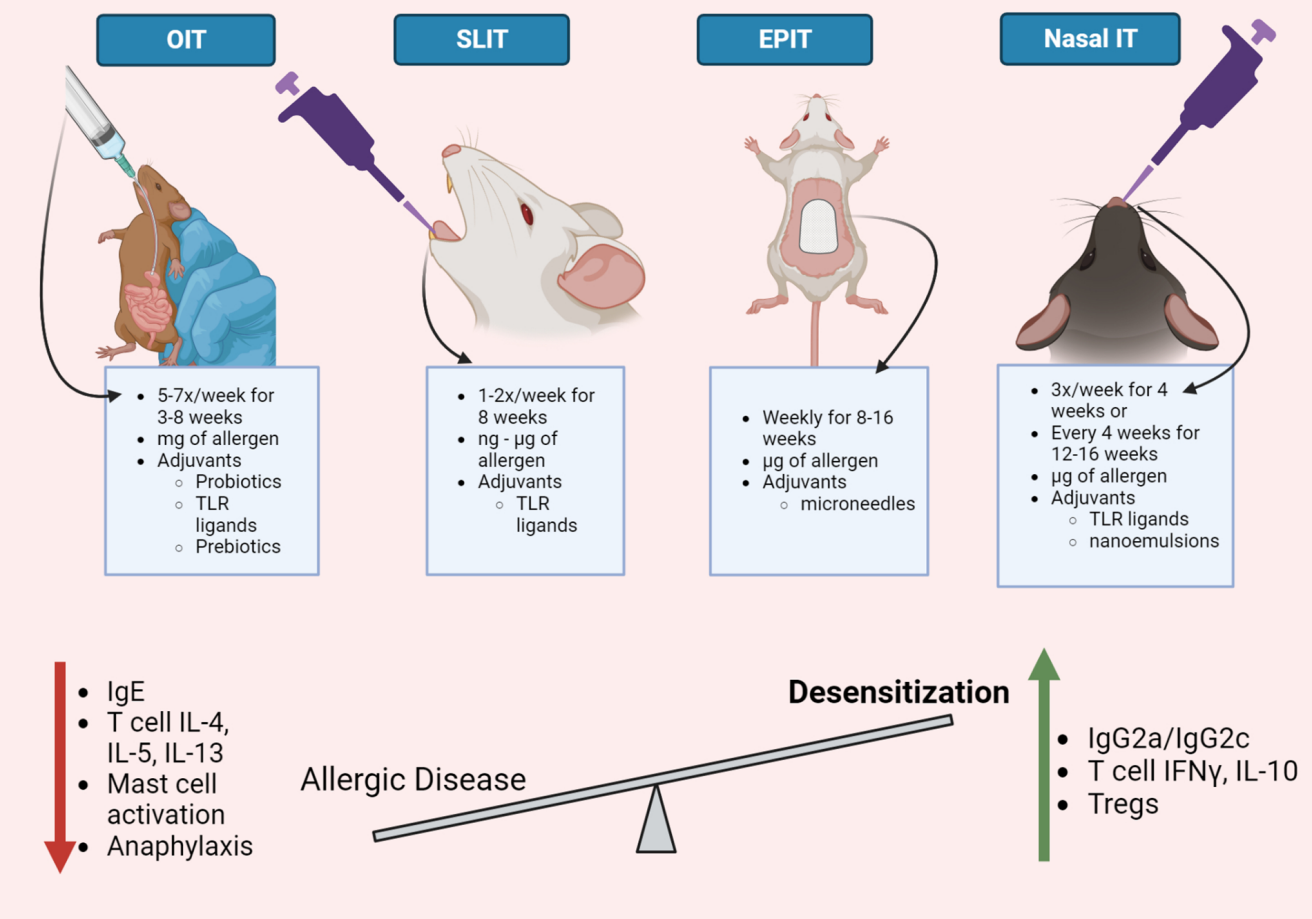
Immunotherapy routes for food allergy. Oral, sublingual, epicutaneous, and nasal routes have been evaluated to administer allergen-specific immunotherapy in mouse models of food allergies. Although the experimental details vary between immunotherapy routes, mouse models of allergen-specific immunotherapy demonstrate a reduction in allergic disease severity and an increase in allergen desensitization. Common immunological changes observed in mouse models of allergen-specific immunotherapy include a decrease in allergen-specific IgE and IgG1, Th2 cytokines, mast cell activation, and systemic anaphylaxis. Food allergy immunotherapy also increases allergen-specific IgG2a/c antibodies, T cell production of IFNγ and IL-10, and regulatory T cells. Strategies that enhance desensitization responses in mouse models of food allergy immunotherapy may be useful to improve allergy immunotherapy for human use. This figure was created with BioRender.com.

Preclinical models are beneficial to investigate food allergy immunotherapy because the immune responses that mediate allergies are similar in humans and mice. Like humans, mice must also be sensitized to allergens. Allergen-hypersensitive mice develop similar allergen-specific immune responses as allergic humans, including allergen-specific serum IgE and IgG1 antibodies and Th2-associated cytokines, including IL-4, IL-5, and IL-13 (14–17), and respond to an allergic challenge with enhanced serum mast cell proteases (mmcp), acute hypothermia, or allergic diarrhea (18–20). Mouse models of food allergy provides an opportunity to investigate immunotherapy efficacy without the contribution of genetic diversity and various environmental exposures present in human populations that may influence host responses to immunotherapy. Thus, mouse models are valuable tools to evaluate strategies that modulate allergen-specific immunity and optimize immunotherapy regimens to treat food allergies. Several studies utilize food allergy mouse models to determine the ability of allergen-specific OIT, SLIT, EPIT, and nasal immunotherapy to reduce allergic disease severity (15, 21–23) (Table 1). Allergen-specific immunotherapy administered by various anatomical routes may utilize immunotherapy formulations that include hypoallergenic antigens, immune-modulating adjuvants, and specialized delivery vehicles to induce desensitization (Figure 1). Although several immunotherapy routes and formulations have been used to desensitize allergic mice, the experimental details often vary between studies, which complicates evaluation of experimental immunotherapy variables, including administration route, on food allergy outcomes across published studies. Thus, immunotherapy route comparison studies are required to determine if altering the route of allergen-specific immunotherapy is sufficient to improve the safety and efficacy of allergen-specific immunotherapy or if modified immunotherapy formulations are required to increase immunotherapy efficacy. This review will discuss the experimental details, including immunotherapy route, schedule, and formulation, reported in mouse models of allergen-specific immunotherapy, and their ability to induce allergen-specific immunity that correlate with immunotherapy efficacy to identify immunological endpoints that may be used to improve clinical allergen-specific immunotherapy administered by various anatomical routes.

**Table 1:** Preclinical models of food allergy immunotherapy.

Immunotherapy route	Food allergy model	Immunotherapy schedule	Allergen dose	Formulation	Allergic response outcome	Ref
Oral immunotherapy						
	Peanut	5x/week for 3 weeks	15 mg	PBS	• Decrease hypothermia in C3H mice • Increase mmcp1 in BALB/c mice • Decrease MLN IL-13 and IL-5 in BALB/c mice	(22)
	Egg (ovomucoid, OM)	Daily for 10 days	0.5–5 mg	Water	• Increase vascular permeability • Increase serum IgG1 and IgE • Increase ConA-stimulated IL-4 and IL-5 • Decrease ConA-stimulated IL-10 and IFNγ	(24)
	Egg white (EW)	Daily for 10 days	1–16 mg	Saline	• Decrease egg white-specific IFNγ • Increase OM-specific plasma IgE • Increase EW and OM-specific IgA	(25)
	Peanut	Daily for 8 weeks	1–5 mg	PBS	• Decrease serum IgE • Increase serum IgG2a • Decrease peanut-specific IL-5 and IL-13 • Increase peanut-specific IL-10 • Increase Foxp3+ T cells	(26)
	Egg ovomucoid (OM)	Continuous feeding for 4 weeks	1% OM	Mouse chow containing 19% casein + 1% OM	• Reduced allergy symptoms after an oral challenge • Reduced vascular permeability • Increase plasma OM-specific IgA	(27)
	Egg white	Continuous feeding for 4 weeks	0.01%, 0.1%, or 1% EW	Mouse chow containing casein	• 1% EW reduced diarrhea incidence • Increase OM-specific IgE and IgA after 2 weeks of therapy but decrease IgE and IgA at the end of the study • Increase EW-specific IFNγ, IL-10 and Foxp3+ T cells	(28)
	Buckwheat	Continuous feeding for 6 weeks	0.03% phosphorylated or unphosphorylated antigen in mouse feed	Mouse chow	• Phosphorylated antigen reduced allergic symptom score • Decreased total and allergen-specific serum IgE • Increased total and allergen-specific serum IgA • Increased Tfh cells in Peyer's patches	(29)
	Egg white	3x/week for 3 weeks	5 mg of digested protein	In PBS	• Induce DCs to suppress T cell IL-5, IFNγ, and IL-17 in coculture systems • Induce DCs and T cell cocultures to increase TGFβ and Foxp3+ RORγt+ cell populations	(30)
	Egg ovomucoid	Daily for 2 weeks	1–50 mg	Lactobacillus casei rhamnosus (1 × 10^9^ CFU per day)	• Prevented allergen-induced hypothermia • Decreased allergy symptom score • Decreased serum IgE, IgA, IgG1, and IgG2a compared to OIT alone • Reduced goblet cell numbers in the small intestines	(31)
	Peanut	Once a week for 4 weeks	200 μg	In PLGA nanoparticles +CpG (1.8 μg)	• Reduced allergen-induced hypothermia • Decreased allergic symptom scores • Decrease plasma histamine • Decrease serum peanut-specific IgE and IgG1 • Increase serum peanut-specific IgG2a • Decrease peanut-specific IL-4, IL-5, and IL-13 • Increase peanut-specific IFNγ	(32)
	Peanut	5x/week for 3 weeks	1.5 or 15 mg	OIT in PBS while continuously feeding a diet containing 1% short and long-chain fatty acid fructooligosaccharide prebiotics	• High dose OIT reduces hypothermia in the presence and absence of prebiotics • Low dose OIT reduces hypothermia in the presence of prebiotics	(33)
	Cow milk	5x/week for 3 weeks	10 mg	OIT in PBS supplemented with 0.6 g/kg bodyweight per day of sodium butyrate	• Suppresses mast cell activation and mast cell IL-13 and IL-6 production • Reduce allergic symptoms after intragastric and intradermal challenge • Decrease allergen-specific serum IgE	(34)
Sublingual immunotherapy						
	Peach	Once a week for 8 weeks	1 nmol	With CpG (50 μg) in PBS	• Decrease hypothermia • Decrease serum IgG1 and IgE • Increase Tregs • Decrease CD4+IL-4+ cells • Increase CD4+IFNγ+ and CD4+IL-10+ cells	(23)
	Peanut	Once a week for 8 weeks	100 μg	1.2% carboxymethylcellulose	• Decrease serum IgE • Increase serum IgG2a • Decrease peanut-specific IL-5 and IL-13 • Increase peanut-specific IL-10 • Increase Foxp3+ T cells	(26)
	Cow milk	Twice a week for 8 weeks	10 pg–10 ng	Omp16 (50 μg)	• Reduced allergy clinical symptom score and foot pad inflammation compared to SLIT alone • Increase antigen-specific IgG2a • Decrease serum IgG1:IgG2a ratio • Decrease antigen-specific IL-5 and IL-13 • Increase antigen-specific IFNγ • Increase IFNγ+ cells in both CD4+ and CD8+ cells that transfer protection to other hypersensitive mice	(35)
	Peach	Once a week for 8 weeks	1, 2, or 5 nmol	PBS	• 2 nmol dose prevented allergen-induced hypothermia 1 and 5-weeks post-therapy • Decrease CD4+IL-4+ cells • Increase CD4+IFNγ+ cells • Increase in Tregs and CD4+IL-10+ cells	(36)
Epicutaneous immunotherapy						
	Cashew	48-h patch every week for 8, 12, or 16 weeks	50 μg	Phosphate buffer	• Decrease allergy symptoms and hypothermia after 16-week therapy • Reduce mmcp1 after 8-week therapy • Reduce IgE and IgG1 after 16-week therapy • Increase IgG2a	(21)
	Peanut	48-h patch every week for 8 weeks			• Decrease serum IgE • Increase serum IgG2a • Decrease peanut-specific IL-5 and IL-13 • Increase Foxp3+ T cells • Increase Foxp3+ T cells from naïve population • Maintain enhanced Treg population 8 weeks post-therapy	(26)
	Peanut	Weekly for 5 weeks	∼11.3 μg for microneedles 100 μg for EPIT patch	Microneedles with a 5-min exposure time EPIT patch with 24-h exposure time	• Microneedles reduced allergy symptom score and serum mmcp1 levels • Microneedles prevented allergen-induced hypothermia • Microneedles decreased antigen-specific IL-4, IL-5 and IL-21 in the spleen and MLN • Microneedles increased antigen-specific IFNγ in the spleen and MLN • Microneedles increased antigen-specific IL-10 in the MLN. Microneedles enhance serum IgG2a and IgG2b and decrease serum IgE compared to EPIT	(37)
Nasal Immunotherapy						
	Peanut	3x/week for 4 weeks	40 μg	CpG (20 μg) in saline	• Decreased allergic symptoms and hypothermia, Decrease IL-5 and IL-13 • Increase IL-10 and IFNγ • Increase serum IgG2c and mucosal IgA	(15)
	Cow milk	Every 4 weeks for 16 weeks	20 μg	20% Nanoemulsion	• Reduced allergen-induced hypothermia, clinical symptom scores and serum mmcp1 4 and 16 weeks post-therapy • Decreased IL-4 and IL-13 and increased IFNγ,IL-10, IL-17, and IL-22 4-weeks after therapy. • Further enhanced immune responses compared to nasal therapy with antigen alone 16 weeks post-therapy • Decreased serum IgE and IgG1 and increased serum IgG2a	(38)
	Peanut	Every 4 weeks for 12 weeks	20 μg	20% Nanoemulsion	• Decrease antigen-specific serum IgE and IgG1 • Increase antigen-specific serum IgG2a and IgG2b, mucosal IgA • Decrease antigen-specific IL-4 and IL-13 • Increase antigen-specific IFNγ, IL-17, IL-10, and IL-2 • Reduce allergic symptom score and antigen-induced serum mmcp1	(39)

## Preclinical models of food allergy OIT

Oral immunotherapy is the most effective route to induce desensitization in humans, but AEs are a concern; therefore, animal models of OIT are investigating strategies to improve OIT safety. Mouse OIT is often administered by gastric gavage to deliver the therapy into the stomach. To ensure sufficient allergen amounts reach the intestinal immunological sites, OIT usually delivers milligrams of allergens to hypersensitive mice as frequently as five to seven times per week (22, 26). The duration of mouse OIT regimens can also range from days to weeks. A ten-day OIT regimen evaluated the feasibility of rush OIT to induce allergen-desensitization in ovomucoid (OM)-sensitized mice (24). Oral delivery of increasing OM doses, ranging from 0.5–5 mg, did not improve allergic disease but enhanced allergen-sensitization responses, including total IgE, OM-specific IgG1, and vascular permeability (24). 5 mg of allergen may be suboptimal to induce desensitization using rush OIT, but increasing the allergen dose to 16 mg was also ineffective (25). A ten-day rush OIT may not allow sufficient time to induce allergen desensitization, and a longer immunotherapy regimen may be more effective. A three-week peanut OIT regimen that delivered 15 mg of peanut five times a week reduces peanut-induced hypothermia after a systemic challenge in peanut-hypersensitive C3H mice but enhances serum mmcp1 after a gastric peanut challenge in BALB/c mice (22). The variability in OIT efficacy observed in BALB/c and C3H mice suggests that genetic differences may influence host responses to immunotherapy; however, an ideal OIT regimen would induce protective immunity in multiple mouse strains. Nonetheless, both BALB/c and C3H mice displayed modified allergen-specific serum antibody responses and decreased regulatory T cell (Treg) numbers in the spleen and mesenteric lymph nodes (MLN) after therapy, demonstrating that a three-week OIT regimen is sufficient to induce immune-modulation in both mouse strains despite only protecting one mouse strain from disease (22). Extending OIT beyond three weeks may provide additional immune modulation that improves allergic disease. In contrast to a three-week OIT regimen that increased peanut-specific IgE (22), an eight-week OIT regimen observed decreased peanut-specific IgE (26), which supports human studies that report initial IgE increases that resolve with longer therapy (40). Short OIT regimens may be improved by continuous allergen exposure, as demonstrated by reduced allergic disease and vascular permeability in OM-hypersensitive mice that consumed allergen-supplemented feed for four weeks (27). Continuous allergen feeding increases allergen-specific IgA but does not change allergen-specific IgG. Protection against allergic disease depends on the allergen dose, as only animal feed containing 1% but not 0.1 or 0.01% of the allergen improved allergic disease severity (28). Continuous feeding is more effective for inducing oral tolerance to food antigens than gastric gavage (41); however, continuous allergen feeding may not be practical for human therapy. Thus, alternative strategies beyond expanding allergen exposures are required to improve OIT for human use.

OIT with hypoallergenic antigens may induce desensitization without adverse reactions to improve OIT safety. Adding post-translational modifications to allergens is one mechanism to generate hypoallergenic proteins. A mouse model of buckwheat allergy demonstrated reduced allergic symptoms with OIT performed by continuous exposure to animal feed containing 0.03% phosphorylated allergens (29). Phosphorylated allergens increased local T follicular helper (Tfh) cells and IL-6-producing dendritic cells (DCs) and decreased IL-4 in the Peyer's patches (29), which may support the decreased IgE and enhanced IgA responses also observed after OIT. Fragmenting allergens into peptides can remove IgE epitopes but maintain other immune-modulatory epitopes that induce protective immunity. Pepsin-digested ovalbumin (OP) decreases Th2 cells and increases Treg cells in the lamina propria and MLN in a mouse model of egg white OIT (30). Modulation of local T cells may suppress inflammatory responses in the GI tract. Interestingly, OP OIT also decreases intestinal epithelial lymphoid cell IL-33 production (30), thereby dampening an innate inflammatory response that contributes to allergen sensitization (42). Hence, including hypoallergenic antigens in OIT formulations may reduce AEs while inducing desensitizing immunity.

Exogenous adjuvants can enhance, modulate, or accelerate allergen-specific immune responses to enhance protective immunity when included in immunotherapy formulations (43). Probiotics are healthy bacteria that may reduce local inflammation while providing immune-stimulatory activities that lead to allergen desensitization. OM-OIT performed in the presence of *Lactobacillus rhamnosus* probiotics further prevents allergen-induced hypothermia achieved by OIT with OM alone in OM-hypersensitive mice (31). Probiotics provide adjuvant activity that reduces OM-specific IgA, IgG1, IgG2a, and IgE compared to allergen-alone OIT, which suggests adjuvant-induced immuno-suppression. Immunotherapy adjuvants can also modulate allergen-specific immunity. An OIT formulation containing peanut (200 μg) and CpG (1.8 μg) encapsulated in poly(lactic-co-glycolic acid) (PLGA) nanoparticles that reduces allergic disease also decreases peanut-specific IgE and IgG1 and increases peanut-specific IgG2a (32), demonstrating a shift in serum antibody responses. Increased peanut-specific IgG2a may be due to CpG-enhancing Th1-associated immunity, further supported by the decrease in peanut-specific Th2 cytokines and the increase in peanut-specific IFNγ observed after CpG-adjuvanted OIT (32). Instead of suppressing allergen-specific immunity, CpG-adjuvanted OIT may reduce allergic disease severity by enhancing blocking antibodies that decrease IgE-mediated allergic disease severity, as allergen-specific IgG antibodies are reported to block mast cell-mediated disease (44). It is important to note that the protective effects of CpG-adjuvanted OIT persisted for 16 weeks post-therapy, which supports OIT-induced SU. Adjuvants that increase OIT efficacy with low doses may improve OIT safety. Fructo-oligosaccharide prebiotics allow peanut OIT to prevent hypothermia using a suboptimal allergen dose that requires 10-fold more allergen to protect against anaphylaxis alone (33). Prebiotic-adjuvanted OIT induces local Tregs that suppress mast cell activation (34) and may influence gastrointestinal inflammation to enhance OIT efficacy. Therefore, adjuvanted-OIT may be an effective strategy to maintain OIT efficacy while decreasing AEs.

## Preclinical models of food allergy SLIT

Preclinical food allergy SLIT models demonstrate the sublingual route is an effective route to induce allergen desensitization. SLIT is performed by administering the allergen under the tongue of anesthetized animals to allow sufficient contact time with the sublingual mucosa. SLIT regimens often require at least eight weeks to induce desensitization, as a four-week immunotherapy regimen did not prevent anaphylaxis in milk-hypersensitive mice (35). However, extending immunotherapy for four more weeks significantly decreased the severity of allergic reactions (35). Notably, the allergen dose increased 1,000-fold during the last four weeks, which may have contributed to SLIT-induced protection. Some SLIT models administer immunotherapy once a week (23, 36), while other studies deliver two doses a week (35), but different immunotherapy formulations complicate comparing the effect of one vs. two doses per week on SLIT efficacy. However, SLIT conditions that induce desensitization with minimal allergen doses may be more desirable.

SLIT can induce desensitization with lower allergen doses than OIT, which may reduce the AEs observed during OIT. SLIT containing 10 pg–10 ng of milk allergen induces desensitization in hypersensitive mice (35), which is 1,000,000-fold less allergen than milk OIT studies that administer 10 mg (34). Low-dose SLIT reduced allergic symptom scores and decreased allergen-specific IgE and IL-13 (35), demonstrating the ability of low allergen doses to modulate immune responses to protect against allergic disease. SLIT performed with peptide antigens can protect against food allergies without the risk of IgE-mediated AEs. T cell epitopes from the peach allergen Prup3 were used to develop glycodendropeptide antigens that contained mannose dendrons in a SLIT formulation (36). Glycodendropeptides enhance allergen uptake through interactions between the mannose molecules on the allergen and DC C-type lectin receptor (45), which may allow lower allergen doses to modulate host immune responses. SLIT containing 2 nmol of glycodendropeptides prevented allergen-mediated anaphylaxis that was maintained for at least five weeks post-immunotherapy (36), suggesting effective SLIT may also induce SU. Tregs are potential SU mediators, as increased CD4+CD25+Foxp3+ and CD4+IL-10+ cells are observed after therapy (36). Different Treg methylation patterns have been observed in Prup3-hypersensitive mice that remained sensitized or achieved desensitization or SU (46), supporting the idea that SLIT influences Tregs in allergic disease outcomes.

Incorporating adjuvants with SLIT can enhance allergen-protective immunity. *Brucella abortus* outer membrane protein 16 (Omp16)-adjuvanted milk SLIT reduced allergic disease compared to SLIT with milk alone in hypersensitive mice (35). Although milk SLIT decreases serum IgE and splenic IL-5 and IL-13 and increases serum IgG2a and splenic IFNγ, OMP16-adjuvanted SLIT further enhances the shift from allergen-specific Th2 immunity towards Th1. An increase in IFNγ-producing CD4+ and CD8+ T cells, observed after OMP-16-adjuvanted SLIT, transferred protection to milk-sensitized mice, while only CD4+IFNγ+ cells from milk-treated mice were protective (35). Omp16 may improve immunotherapy efficacy by activating additional cellular populations to suppress allergic responses. CpG is another adjuvant used in SLIT. CpG-adjuvanted Prup3 SLIT prevents allergen-induced hypothermia and decreases allergen-specific serum IgE, IgG1, and T cell proliferation (23). Enhanced T cell-specific IFNγ and IL-10 were observed in Prup3-hypersensitive mice that obtained desensitization and may suppress antigen-induced T cell proliferation. CpG-adjuvanted SLIT utilized 50 μg of CpG, which is 20-fold more than OIT studies (32); however, SLIT utilized 1 nmol of allergen while OIT required 200 μg of allergen to induce desensitization. Therefore, adjuvanted-SLIT may be an effective strategy to generate allergy-suppressing immunity using a lower allergen dose that maintains an enhanced safety profile compared to OIT.

## Preclinical models of food allergy EPIT

Skin exposure is hypothesized as a natural method of food allergen-sensitization in humans (47); therefore, allergen-immunotherapy administered via the skin may be an effective strategy to reverse food allergies. Animal EPIT models administer allergen-coated patches to mouse skin for hours, allowing the allergen to absorb across the stratum corneum to antigen-presenting cells in the underlying epidermis (48). Cashew EPIT that applied 50 μg of allergen to shaved mouse skin for 48 h every week reduces serum mmcp after eight weeks of therapy but requires 16 weeks of treatment to prevent cashew-induced anaphylaxis (21), suggesting immunotherapy duration influences EPIT efficacy.

Mechanical adjuvants accelerate desensitization induced by cutaneous immunotherapy. Allergen-coated microneedles penetrate the dermis and may enhance antigen trafficking to lymphoid tissues (37). Peanut-coated microneedles administered for five minutes delivered ∼11.3 μg of peanut to peanut-hypersensitive and reduced hypothermia and serum mmcp1 compared to 100 μg of peanut in EPIT patches after a 24-h exposure (37). Peanut-EPIT, performed for five weeks, was less effective than cashew-EPIT that was performed for 16 weeks (21), and the immunotherapy duration may account for the decreased efficacy of peanut-EPIT (37). The authors note that longer peanut-EPIT regimens suppress peanut allergies; however, microneedle immunotherapy accelerated desensitization to induce allergy suppression in three weeks (37). Increased Th1 and Treg-associated responses were observed after cutaneous microneedle immunotherapy (37), and several studies also demonstrated increased Treg responses immediately and eight weeks post-EPIT, suggesting SU (26, 49). EPIT-induced Tregs display a hypomethylation pattern that may mediate SU (50), and in the absence of Tregs, EPIT fails to reduce allergen-specific Th2 cytokines, IgE, and mediate allergy protection (49). Thus, microneedle EPIT may be a mechanism to induce SU in humans and increase the attractiveness of EPIT as an alternative to OIT.

## Preclinical models of food allergy nasal immunotherapy

Nasal antigen exposure can lead to immunological tolerance (51); however, concerns about severe respiratory or central nervous system reactions (52, 53) may reduce the enthusiasm for allergen-specific nasal immunotherapy. Nasal immunotherapy formulations must be carefully designed to induce desensitization without AEs. Nasal immunotherapy administers low allergen doses through the animal's nostrils in small volumes, reducing the risk of allergen exposure in the lower respiratory tract. Formulating allergens for nasal delivery in mucoadhesive vehicles may also maintain the allergen in the nasal cavity (54). Specialized formulations such as nanoparticles may encapsulate allergens and promote rapid cellular uptake (55), bypassing antibody-coated granulocytes that contribute to inflammatory responses to ensure the safety of nasal allergen immunotherapy.

Nasal immunotherapy induces desensitization in food allergen-hypersensitive mice with fewer allergen doses than other immunotherapy routes. Nasal immunotherapy administered three times a week for four weeks reduced the severity of peanut-induced anaphylaxis in peanut-hypersensitive mice (15), while some OIT, EPIT, and SLIT studies required eight weeks to induce desensitization (21, 26, 35). Although shorter OIT animal studies are reported, these studies often administer up to five doses per week for three weeks (22), which increases the total number of allergen exposures. Nasal milk immunotherapy administered every four weeks for four administrations has also been reported to reduce systemic anaphylaxis four and 16 weeks post-immunotherapy, demonstrating SU (38). It is possible that the milk-hypersensitive mice naturally lost sensitization because acquired tolerance to milk is reported in humans (56); however, control animals demonstrated sensitization was maintained. Thus, immunotherapy that utilizes a four-week interval to reduce allergies is an improvement upon OIT that requires daily administrations (57) and may improve patient quality of life.

Food allergy nasal immunotherapy often contains vaccine adjuvants to alter sensitization-induced immune responses. Peanut-hypersensitive mice nasally exposed to 40 μg of peanut develop enhanced peanut-specific serum IgG and mucosal IgA; however, the addition of CpG (20 μg) further increases peanut-specific serum IgG2c and mucosal IgA compared to peanut alone (15) confirming the adjuvant activity of CpG observed in SLIT and OIT (23, 32). CpG-adjuvanted peanut nasal immunotherapy reduced the severity of systemic anaphylaxis and decreased Th2 cytokines while increasing IFNγ and IL-10 (15). A shift from peanut-induced Th2 immunity towards Th1 and Treg immunity was observed after nasal immunotherapy with a nanoemulsion-adjuvanted immunotherapy formulation containing 20 μg of peanut in a 20% nanoemulsion mixture (39). The shift in T cell responses may occur by Th2 cells acquiring a new phenotype or enhancing a new population of T cells that may suppress the Th2 immunity induced by sensitization. Increased IFNγ in T cells that previously produced IL-13 and increased IL-10 in cells that never made IL-13 were observed in peanut-desensitized mice (15). The increase in IL-10-producing T cells suggests nasal immunotherapy increases Tregs, and IL-10 may be a key regulator of allergies as allergic disease severity rises in the absence of IL-10 (58). Adjuvanted allergen-specific nasal immunotherapy can also modulate immune responses in distal mucosal sites by decreasing jejunum mast cells after repeated oral challenge (38) and down-regulating intestinal ILC2 responses in animals with hypersensitivity to multiple food allergens (59), which may allow allergen-specific nasal immunotherapy to suppress immunity to bystander allergens (59). Thus, adequately formulated nasal immunotherapy effectively reduce allergic disease in mice and may be an effective immunotherapy strategy in humans.

## Concluding remarks

Allergen-specific immunotherapy administered by several anatomical routes has been evaluated to treat human food allergies; however, immunotherapy safety and efficacy remain a concern. Preclinical models of food allergy have identified strategies to improve the limitations of allergen-specific immunotherapy, including hypoallergenic antigens, alternative delivery vehicles, and vaccine adjuvants. Future clinical studies should evaluate strategies that improve immunotherapy efficacy in preclinical models to determine if alternative immunotherapy formulations enhance the safety and effectiveness of OIT, SLIT, EPIT, and nasal immunotherapy. The results from animal allergen immunotherapy studies may identify biomarkers that indicate immunotherapy outcomes. Preclinical food allergy immunotherapy studies have identified modifications in allergen-specific immune responses in mice with reduced allergic disease severity, including enhanced mucosal IgA, a shift in T cell responses, and decreased mast cell activation. The immune responses observed in desensitized animals that complete immunotherapy may be potential checkpoints in clinical studies to monitor immunotherapy efficacy. Lastly, the variability in allergen-specific immunotherapy efficacy observed in animals that receive the same treatment (15, 21, 32, 35) provides an opportunity to study immunotherapy responders and non-responders, which may elucidate immune targets in humans that can be modified to improve the effectiveness of allergen-specific immunotherapy. The information gained from preclinical food allergy immunotherapy studies will be instrumental in developing more effective allergen-specific immunotherapy regimens in humans.
